# Molecular Dynamic Simulation Insights into the Normal State and Restoration of p53 Function

**DOI:** 10.3390/ijms13089709

**Published:** 2012-08-03

**Authors:** Ting Fu, Hanyi Min, Yong Xu, Jianzhong Chen, Guohui Li

**Affiliations:** 1 Laboratory of Molecular Modeling and Design, State key Laboratory of Molecular Reaction Dynamics, Dalian Institute of Chemical Physics, Chinese Academy of Sciences, 457 Zhongshan Road, Dalian 116023, China; E-Mail: futing@dicp.ac.cn; 2 Department of Bioscience and Biotechnology, Dalian University of Technology, Dalian 116024, China; 3 Graduate University of Chinese Academy of Sciences, Beijing 100049, China; 4 Department of Ophthalmology, Peking Union Medical College Hospital, Chinese Academy of Medical Sciences & Peking Union Medical College, Beijing 100730, China; E-Mail: wredge@sohu.com; 5 Institute of Chemical Biology, Guangzhou Institutes of Biomedicine and Health, Chinese Academy of Sciences, 190 Kaiyuan Avenue, Guangzhou 510530, China; E-Mail: xu_yong@gibh.ac.cn; 6 Department of Mathematics and Physics, Shandong Jiaotong University, Jinan 250031, China; E-Mail: chenjianzhong1970@163.com

**Keywords:** p53, MDM2, MDMX, molecular dynamic simulation, conformational change, protein-protein interaction, protein-ligand interaction

## Abstract

As a tumor suppressor protein, p53 plays a crucial role in the cell cycle and in cancer prevention. Almost 50 percent of all human malignant tumors are closely related to a deletion or mutation in p53. The activity of p53 is inhibited by over-active celluar antagonists, especially by the over-expression of the negative regulators MDM2 and MDMX. Protein-protein interactions, or post-translational modifications of the C-terminal negative regulatory domain of p53, also regulate its tumor suppressor activity. Restoration of p53 function through peptide and small molecular inhibitors has become a promising strategy for novel anti-cancer drug design and development. Molecular dynamics simulations have been extensively applied to investigate the conformation changes of p53 induced by protein-protein interactions and protein-ligand interactions, including peptide and small molecular inhibitors. This review focuses on the latest MD simulation research, to provide an overview of the current understanding of interactions between p53 and its partners at an atomic level.

## 1. Introduction

p53 is a tumor suppressor protein, encoded by the P53 gene initially reported in 1979 [1]. p53 is commonly referred to as “the guardian of the genome”, as it plays a vitally important role in multi-cellular organisms, where it regulates the cell cycle (promoting cell apoptosis, maintaining genomic stability and inhibiting tumor angiogenesis) and functions as a tumor suppressor involved in slowing or monitoring the cell division and preventing genome mutation in normal circumstances [2–5]. When cells are damaged, p53 determines the degree of DNA damage and either promotes cellular repair when the degree of damage is not severe, and cell apoptosis when the damage is too severe to be repaired. Damaged cells with a P53 deficiency do not have this functionality and will continue to begin uncontrolled division even in adverse conditions. Under normal circumstances, p53 is strictly controlled at minimal levels through a fast continuous degradation mechanism with a half-life of only about 20 min. In abnormal conditions, however, p53 with extended half-life is stabilized and activated in response to stressful stimuli such as DNA damage, ionizing radiation, UV-radiation, heat shock, nitric oxide, hypoxia and oncogene activation [6–8].

**Figure 1:**
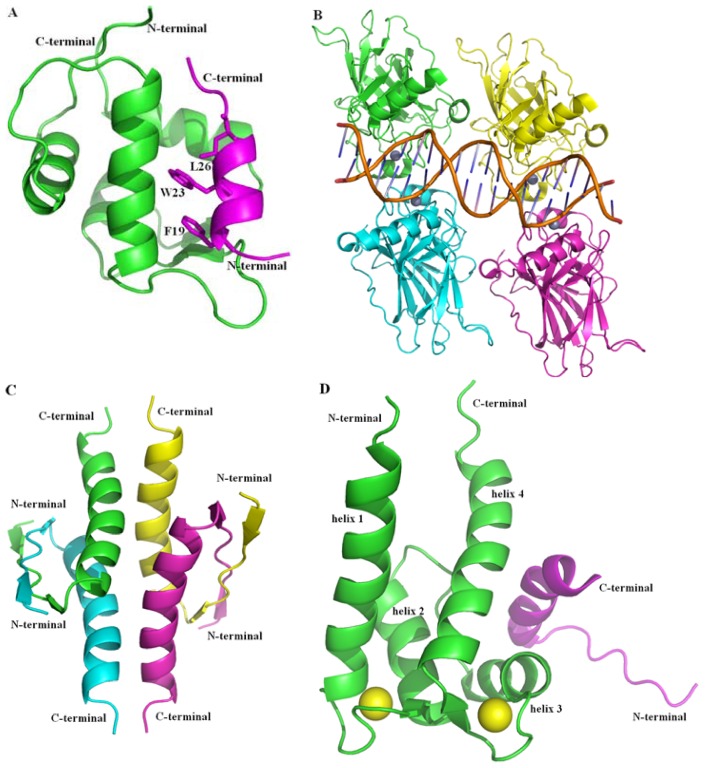
Structures of p53 protein. (**A**) The complex of p53 transcriptional activation domain (TAD) fragment bound to MDM2 (PDB 1YCR) [18] is shown in cartoon, p53 TAD fragment (residues 17–29) is shown in magenta and the three most important residues are shown in stick, MDM2 (residues 25–109) is shown in green; (**B**) The tetramer of the DBD of p53 (PDB 3KMD) [15] is shown in cartoon and the four monomers (residues 92–291) are colored in green, cyan, magenta and yellow, respectively; Zn^2+^ is shown in sphere and dirtyviolet, and the DNA is shown in stick; (**C**) The tetramer of oligomerization domain (OD) of p53 (PDB 1PES) [16] is shown in cartoon and the four monomers (residues 325–355) are colored in green, cyan, magenta and yellow, respectively; (**D**) The complex of p53 C-terminal regulatory domain (CTD) fragment bound to S100 calcium-binding protein B (PDB 1DT7) [19] is shown in cartoon, p53 CTD fragment (residues 377–387) is shown in magenta and yellow, S100B (residues 1–91) is shown in green and cyan and the two Ca^2+^ are shown in sphere and are colored in, consistent with the S100B protein for the two subunits, respectively. Figures were created with Pymol (http://pymol.org) [20].

p53 is a homo-tetramer, with each flexible monomer containing 393 residues that can be divided into several regions, as follows [9–11]: The N-terminal transcriptional activation domain (TAD) includes an acidic activation domain 1 (AD1, residues: 1–42) and domain 2 (AD2, residues: 43–62). This region is a highly charged area including a large number of acidic amino acid residues and phosphorylation sites, and is involved in the regulation of apoptotic activity. The adjacent proline-rich domain at residues 63–97, is essential for the apoptotic activity of p53. The core DNA-binding domain (DBD, residues: 102–292), contains one zinc atom and several electropositive arginine amino acids which interact with DNA. This region can bind to sequence-specific DNA, and is often unstable and contains most of the inactivating single missense mutations occurring in human tumors [12]. The nuclear localization signaling domain at residues 316–325, is involved in the intracellular localization of p53. The oligomerization domain (OD, residues: 326–356) is responsible for tetramerization which is essential for the activity of p53 *in vivo*. The intrinsically disordered C-terminal regulatory domain (CTD), a flexible region is involved in down-regulation of the central DNA binding domain at residues 363–393. The three-dimensional structures of the entire DNA-binding domain [13–15] (PDB ID: 3KMD, Figure 1B) [15] and oligomerization domain (PDB ID: 1PES, Figure 1C) [16] have been solved while the structures of the N-terminal transcriptional activation domain and C-terminal regulatory domain have not been determined (Figure 1). Bell *et al.* [17] argued that full-length wild-type p53 protein contains large unstructured regions in its N- and C-terminal domains, is destabilized and easy to unfold and loses its biological activities in the absence of modifications or stabilizing partners. The three-dimensional structures of p53 TAD fragment bound to MDM2 (PDB ID: 1YCR, Figure 1A) [18] and p53 CTD fragment bound to S100 calcium-binding protein B (PDB 1DT7, Figure 1D) [19] are shown in Figure 1. All the figures were created with Pymol [20].

It is clear that the stability and transcriptional activity of p53 are regulated through a complex cascade of post-translational modifications, such as phosphorylation (the 17 known phosphorylation sites in human p53 are Ser6, Ser9, Ser15, Thr18, Ser20, Ser33, Ser37, Ser46, Thr55, Thr81, Ser149, Ser150, Ser155, Ser315, Ser376, Ser378 and Ser392), and acetylation of critical lysines (AcLys382), methylation (MeLys382) and ubiquitination [21–24]. Furthermore, the destabilized structure may allow the physiological interaction of p53 with numerous protein partners and regulation of its turnover [14]. Many biological, structural, mutagenesis and computational studies showed that the pro-apoptotic activity of p53 is complicated, and affected by protein-protein interactions [25,26]. For example, the TAD fragment of p53 involving residues 12–26, has high probability of forming a short α-helix that is capable of interacting with protein partners, such as the transformed mouse 3T3 cell double minute 2 (MDM2, or HDM2 for the human congener, PDB ID: 1YCR, Figure 1A) [18] and MDM2-related protein (MDMX, also named MDM4) [27]. As a negative regulator, MDM2/X can induce inactivation of over-expressed p53 in a normal cell. In addition to the key regulators MDM2 and MDMX which interact with the target p53 through TAD, some other partners have been found in recent years. Bcl-XL, one member of the Bcl-2 family proteins, is identified as a binding target of p53 via TAD and results in transcription-independent apoptotic activity [28–30]. Azurin, a copper-containing protein with electron transfer activity, has been reported to bind p53 via either the TAD or the DBD domains of p53 [31–33]. The single-stranded DNA-binding protein, replication protein A (RPA) (PDB ID: 2G3B) [34] and the RNA polymerase II transcription factor B subunit 1 are also found to interact with p53 TAD (PDB ID: 2GS0) [35]. The DBD of p53 is mainly responsible for sequence-specific DNA binding (PDB ID: 3KMD, Figure 1B) [15] and some protein-protein interactions. The large T-antigen of Simian Virus 40 binds to DBD and induces the dramatic conformational changes at the DBD of p53 (PDB ID: 2H1L) [36]. Moreover, the extreme CTD not only binds to DNA and RNA sequences, but also is critical for regulation of p53 function [37] and is capable of adopting multiple folded conformations upon binding to different partners such as S100 calcium-binding protein B (S100B) (PDB ID: 1DT7, Figure 1D) [19], sirtuin protein (Sir2) (PDB ID: 1MA3) [38], cAMP response element-binding (CREB) binding protein (CBP) (PDB ID: 1JSP) [39], the histone methyltransferase SET9 (also known as SET7/9) (PDB ID: 1XQH) [40] and the cyclin A/cyclin-dependent protein kinase 2 complex (PDB ID: 1H26) [41].

**Figure 2:**
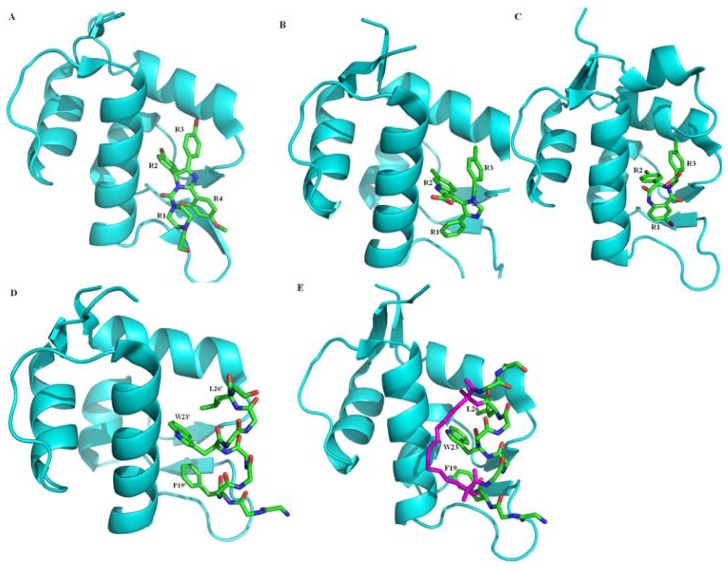
Structures of the complexes of MDM2 with the non-peptide inhibitor and peptide inhibitors. MDM2 are shown in cartoon and cyan, inhibitors are shown in stick and green; (**A**) MDM2 with Nutlin (from PDB 1RV1) [59]; (**B**) MDM2 with K23 (from PDB 3LBK) [45]; (**C**) MDM2 with DIZ (from PDB 1T4E) [60]; (**D**) MDM2 with PMI (from PDB 3LNZ) [49], the parts imitating the three most important residues of p53 (F19′, W23′ and L26′) are presented as stick; (**E**) MDM2 with SAH-p53-8 stapled-peptide (from PDB 3V3B) [61], the all-hydrocarbon staple is shown in stick and magenta. Figures were created with Pymol (http://pymol.org) [20].

Mutation of TP53 is the most common genetic change in human cancers, which mainly occurs in the highly conserved residues, particularly Arg175, Gly245, Arg248, Arg249, Arg273 and Arg282 hot spots. More than 50 percent of all the human malignant tumors, for instance liver cancer, breast cancer, bladder cancer, gastric cancer, colon cancer, prostate cancer, soft tissue sarcoma, ovarian cancer, brain tumor, lymphatic tumors, esophageal cancer, lung cancer, osteogenic sarcoma, are the result of a mutation or deletion of the TP53 gene [42–44]. Due to a conformational change, the mutational TP53 gene loses regulatory control of cell growth, apoptosis and DNA repair. For the other half of human cancers, p53 retains its wild-type form but the activity is greatly reduced due to protein-protein interactions with key regulatory partners, such as MDM2 and MDMX. Therefore, restoring endogenous p53 functions by the disruption of p53-MDM2/X interaction using non-peptide small molecule inhibitors [45,46] or peptide [47–49] currently plays one of the most promising strategies for anti-cancer drug design and development. Several related reviews [46,50–58] have reported, a large number of potent and selective inhibitors that have been identified and can be divided into two categories (Figure 2). One type is the non-peptide small molecule inhibitors, which include but are not limited to, Spiro-oxindoles, piperazine-4-phenyl derivatives, chlorofusin, Cis-imidazolines (nutlins, Figure 2A) [59], imidazole-indoles (K23, Figure 2B) [45], norbornanes, benzodiazepinediones (DIZ, Figure 2C) [60], chromenotriazolopyrimidines, terphenyls, isoindolinones, sulfonamides, chalcones and boronic chalcones. All the above mentioned small molecule inhibitors are used to fill the Trp23 sub-pocket with either a *p*-halogenated phenyl group or a combination of 6-chloro(ox)indole and the Phe19 and Leu26 sub-pockets with multiple hydrophobic interactions. Another type includes peptide inhibitors such as p53-like peptides (PMI, Figure 2D) [49], miniproteins and peptidominetic inhibitors, which mimic the identical hot-spot triad of Phe19, Trp23 and Leu26 of p53. To avoid proteolytic degradation, a series of stabilized alpha-helix of p53 (SAH-p53) cyclic peptides are generated by adopting the “peptide stapling” strategy (Figure 2E) [61]. In addition, S100B has been confirmed to interact with the CTD of p53 protein and inhibit p53 aggregation and down-regulate p53 protein levels and activity [62,63]. Blocking the S100B-p53 interaction and protecting p53 from S100B-dependent down-regulation by ligands is another way to restore the p53 tumor suppressor function [64]. Several other proteins bind to or modify the p53 CTD, for instance, Sir2 specifically binds and deacetylates p53 protein, and activate the tumor suppressor [38].

Computational methods, including molecular docking, molecular dynamics (MD) simulation and binding free energy prediction are usually applied to supplement the experimental studies in order to understand the mechanism at an atomic level or to improve the efficiency of molecular design or discovery. Over the past decade, there has been a dramatic increase in the number of theoretical studies of p53, along with the experimental data and structural information. Previous reviews [50–57] primarily have been concerned with the interactions of p53-MDM2/X and MDM2/X-inhibitors based on the three-dimensional structures available. Many computational studies were carried out to understand p53’s intrinsic flexibility, the molecular mechanism of interactions between p53 and partners, and the conformation changes of p53 induced by protein-protein interactions and protein-ligand interactions, including peptide and small molecular inhibitors. More than 40 articles discussing the dynamic simulations of p53 have been published in the latest three years. The dynamics, flexibility and ligand-induced conformational changes of p53 have not been completely summarized. Additionally, the dynamics characteristic of other domains of p53 has not been reviewed. In this review, the latest MD simulations discussing the protein-protein interactions and the flexibility of native p53 in TAD, DBD, OD and CTD domains, and the inhibitors (small molecule and peptide inhibitors) are discussed.

## 2. Advance in Molecular Simulations

### 2.1. Molecular Docking, MD Simulation and Binding Free Energy Prediction

Molecular docking, a useful computational method for rational drug design, can predict the preferred binding orientation and conformations of small molecule, and the strength of association or binding affinity of the complex through scoring functions. Docking software includes AutoDock, Dock, 3D-Dock, Affinity, LigandFit, FRED, Surflex, HEX, Glide, GOLD, ICM, and MVD as examples. Molecular Dynamics (MD) is a computer simulation method that relies mainly on Newton mechanics to simulate the physical interactions and movement of atoms and molecular systems. To obtain the dynamic characteristics and the understanding of interaction mechanism at an atomistic scale, MD simulation packages like AMBER, CHARMM, GROMACS and NAMD are applied widely in the modeling of biomolecules. Binding free energy prediction has been regarded as a powerful and valuable tool to explore the binding mechanisms and binding affinity of inhibitors to proteins. The molecular mechanics Possion-Boltzmann/generalized Born surface area (MM-PB/GBSA) method has been used successfully in explaining protein-protein and protein-inhibitor interactions [65]. Several computational strategies have been adopted to get a deep insight into the interactions between p53 and its partners.

### 2.2. Analysis of p53-Protein Interaction

#### 2.2.1. Protein-Protein Interaction Located in TAD of p53

**Figure 3:**
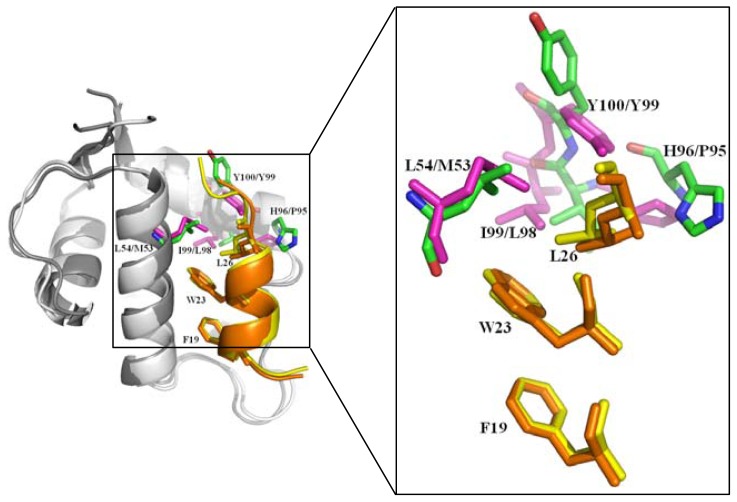
The superimposition of the structures of p53-MDM2 and p53-MDMX complex. The complex of p53-MDM2 (PDB 1YCR) [18] is shown in cartoon, p53 TAD fragment (residues 17–29) is shown in yellow and the three most important residues Phe19, Trp23 and Leu26 are shown in stick, MDM2 (residues 25–109) is shown in white and the four residues Leu54, His96, Ile99 and Tyr100 in MDM2 are shown in green and stick; The complex of p53-MDMX (PDB 3DAB) [27] is shown in cartoon, p53 TAD fragment (residues 17–29) is shown in orange and the three most important residues Phe19, Trp23 and Leu26 are shown in stick, MDMX (residues 23–110) is shown in grey and the equivalent residues Met53, Pro95, Leu98 and Tyr99 in MDMX are shown in light magenta and stick; the spatial orientation of the side chains of Phe19 and Trp23 of p53 are highly similar, but that of Leu26 is different in p53-MDM2 and p53-MDMX complex. (Insert) Magnified view of important residues of p53 and MDM2/X. Figures were created with Pymol (http://pymol.org) [20].

As the principal negative protein regulators of p53, MDM2 binds a short amphipathic α-helix to the TAD domain of p53 (residues 17–29) (Figure 3) [18], and inhibits the transcriptional activity of p53. MDM2 acts as an E3 ubiquitin ligase and is able to target p53 for proteasomal degradation. Since MDM2 is the target gene of p53, the p53-MDM2 system forms a negative feedback loop, which precisely regulates and controls the protein level and activity of p53 [66–69]. MDMX, highly homologous to MDM2 but lacking ubiquitin ligase activity, negatively regulates the transactivation function of p53 via interaction with the p53 TAD domain [70,71]. Studies have shown that the over-expression or hyper-activation of MDM2 and MDMX directly leads to the negative consequences of loss of p53 normal function during the development of nearly half of human cancers [72]. Expressional levels of MDM2/X and the interactions between p53-MDM2/X are important for p53 functionality. The above evidence illustrates that p53 TAD is one of the most pivotal regions in p53 function.

Small lipophilic molecules or p53 peptide analogues, which disrupt or prevent the p53-MDM2/X interaction and restore the p53 tumor suppression, have become an important strategy for anti-cancer drug design and discovery [50,51,73,74]. The stability of p53 TAD fragment and conformational changes due to protein-protein interactions, in particular between p53 and MDM2/X, and protein-ligand interactions, including peptide and small molecular inhibitors have been most extensively studied [58,75–79]. The three-dimensional structure of p53-MDM2 (PDB ID: 1YCR) (Figures 1A and 3) [18] reveals that the peptide segment (Glu17-Asn29) of p53 TAD as an amphipathic α-helix binds to the deep hydrophobic cleft, which is formed by the N-terminal domain of MDM2 (Glu25-Val109) containing of two pairs of α-helices and an anti-parallel β-sheets. The four key residues Phe19, Leu22, Trp23 and Leu26 of p53, and at least residues Leu54/Met53, Leu57/Leu56, Gly58/Gly57, Ile61/Ile60, Met62/Met61, Tyr67/Tyr66, Gln72/Gln71, His73/His72, Val75/Val74, Phe91/Phe90, Val93/Val92, His96/Pro95, Ile99/Leu98 and Tyr100/Tyr99 of MDM2/X play the dominant roles for the p53-MDM2/X interactions. In particular, three key residues Phe19, Trp23, and Leu26 of p53 insert deeply into the hydrophobic cleft of MDM2. Similarly, the crystal structure of the N-terminal domain of human MDMX (Gln23-Ile111) bound to a 15-residue p53 TAD fragment (Ser15-Asn29) was solved at 1.90 Å (PDB ID: 3DAB) [28] (Figure 3). Although the sequence is highly conserved, and the structure and binding mode are very analogous, the MDMX binding pocket is differently shaped and smaller than that of MDM2, due to the side chains of Met53 and Tyr99 (corresponds to Leu46 and Tyr100 in MDM2) protruding into the hydrophobic cleft of MDMX. Analysis of the dynamics reveals that the most flexible region of MDM2 is the hydrophobic cleft, which becomes more wider and stable when bound to p53 while narrower and highly flexible in the absence of p53 [80]. Moreover, based on available structural data of the MDM2/X protein in complex with ligand, including peptide and small molecular inhibitors [45,47,48,59,60], it is possible to understand the detailed molecular interactions between p53 and MDM2/X.

It is well known that p53 TAD in the absence of partners in solution lacks persistent structural order, which is similar to a classical random coil except for a small helix, and contains numerous phosphorylation sites. This intrinsically disordered structural characteristic enables it to interact with multiple protein partners. To explore the native conformational properties, Espinoza-Fonseca and co-workers [76] carried out all-atom MD simulations of the extended p53 TAD fragment (Met1-Ala39) in solution and found that the leucine-rich hydrophobic clusters play an important role in the formation and stabilization of the p53 TAD fragment (Met1-Ala39) globule-like folded structure. Terakawa *et al.* [81] tested diverse structural ensembles of p53 TAD by a systematic multiscale computational method. They used all-atom replica-exchange MD simulations of a coarse-grained model to generate the structural ensemble, and their results showed that the p53 TAD fragment was preferable and formed a kinked structure in the AD1 region and extended conformation in a proline-rich region. Xiong *et al.* [82] experimentally measured the distributions of Ramachandran Ψ angles for this intrinsically disordered p53 TAD and P27S mutation peptide in unbound state using UV resonance Raman spectroscopy, and they also performed explicit solvent simulations to provide detailed views of these conformational distributions. Both experiment and simulations revealed that the number of PPII-like helical conformation content was decreased and the non-PPII content was increased upon P27S mutation. It turns out that binding of MDM2 induces a helical pattern in the fragment Phe19-Leu25 of p53 TAD. Espinoza-Fonseca *et al.* [83] confirmed that the fragment Phe19-Leu22 displayed a stable, transient helical pattern at sub-microsecond periods. Another MD simulation was carried out by Mavinahalli *et al.* [84] using an implicit solvent method to explore the folding ability of the TAD domains of p53, p63 and p73. The binding region folds into a stable helix when p53 TAD peptide is embedded into MDM2. Residues Phe19, Leu22, Trp23, Leu25 and Leu26 constitute a hydrophobic cluster, which controls the kinetics of helix formation. An ionic interaction between Asp20 and Lys24 along with the other three hydrogen bonds between Leu22 and Glu17 (42% occupancy), Phe19-Trp23 (29% occupancy) and Ser20-Lys24 (21% occupancy) are responsible for the stability of the helix. It is known that the helical conformation of the p53 TAD is necessary for optimal binding of p53 to MDM2. It was once believed that [85] the loss of a key hbond between Thr18 and Asp21 would lead to the destabilized helical structure and reduced affinity for MDM2. Mavinahalli *et al.* [84] shows that phosphorylation of Thr18 results in perturbation of this helicity, whereas phosphorylation of Ser20 actually enhances the helical propensity of p53, and the electrostatic interaction of the phosphate and Lys24 play a vital role in the thermodynamic stability of the helix. MD simulations [84], in accord with the earlier findings [86], suggest that upon phosphorylation of either Thr18 or Ser20, its helical structure is still retained but the affinities for MDM2 are reduced. The computational interpretation [86] is that though the important hbond between Thr18 and Asp21 is broken, there are other alternate patterns of hbonds formed to maintain the helicity; a charge-charge repulsion between the negatively charged phosphate group on Thr18 and the anionic patches in the region of the MDM2 surface, result in the weakening p53-MDM2 interactions. This idea has been confirmed by experiment study [87].

From 1999 to the present, p53-MDM2 interactions have been extensively studied using MD simulations. In recent years researchers are paying more attentions to the binding free energy calculations by using the MM-PB/GBSA method, and estimations are in excellent agreement with the experimental results. This has been well reviewed by Lauria *et al.* [79] and will not be discussed in the present paper.

Although MDM2 and MDMX proteins are highly homologous, they display different dynamic behavior. More recently, Dastidar *et al.* [88] emphasized the role of the dynamics of Tyr100, which was recognized as a putative gatekeeper in p53-MDM2 interactions by MD simulations. It appears that the transition of the Tyr100 side chain from “closed” to “open” enlarges the binding site, thus enhancing the binding of MDM2. It is well known that electrostatic complementarity plays a crucial role in the complex formation. The p53 TAD fragment (Glu17-Asn29) has a strong anionic potential with a net −2e charge, in contrast to the largely cationic potential in MDM2 with net charge of +5e while MDMX with a net charge of +1e. The binding affinity of p53-MDMX is much lower than that of p53-MDM2. Then, the same author [89] examined in full atomic detail of the role of electrostatic interaction on the formation of the p53-MDM2 complex by varying the distance of separation between MDM2 and p53. This study reveals that electrostatic interaction controls p53-MDM2 complex formation at long range, while van der Waals interactions of Phe19, Trp23, Leu26 of p53 determine the complex formation at short range.

In order to investigate the conformational events of free and p53-bound states of MDM2 and MDMX, MD simulations were performed by Carotti *et al.* [90]. The analysis of the trajectories shows that the diverse conformations of Tyr99/Tyr100 (MDMX/MDM2) hold crucial roles in affecting the p53-MDM2 and p53-MDMX recognition, and different interactions in the apo and p53-bound states might affect the shape of the p53 binding cleft. The van der Waals interaction was found to be favorable in the p53-MDM2 simulation while the electrostatic interactions mainly dominate in the p53-MDMX simulation. However, the gain in electrostatic energy could not offset the loss of solvation energy, van der Waals interaction between both proteins MDM2/X and Phe19 and Trp23 of p53 was identified as the dominate event in the peptide recognition.

A systematic analysis of single mutations in the 12-residue fragment of the p53 TAD (Q16ETFSDLWKLLP27) and an equivalent phage optimized peptide (12/1, M1PRFMDYWEGLN12) were undertaken by Brown *et al.* [91]. The 10 complexes between MDM2 and the wild type p53 TAD and mutants P27S, P27A, P27T and P27N, and wild type 12/1 and N12S, N12A, N12T, N12P, were studied by using MD simulations to elucidate the differences in the mechanism and thermodynamics of their interactions. The analysis revealed that single residue substitutions dramatically increase the affinity of the MDM2 binding peptides. In addition, the modification of Ser17 in the N-terminal “lid” of MDM2 is expected to regulate the p53-MDM2 binding, such as S17D can promote the binding of p53 to MDM2. A similar study was performed by Dastidar *et al.* [92], where they carried out detailed MD simulations on the p53 binding domain of wild type (WT) MDM2 (residues 1–119), S17D mutant and the phosphorylated Ser17 (pSer17) with the lid in “open” and “closed” conformations to evaluate the effects of Ser17 modification on p53 binding.

Recently, the recognition process between TAD fragment of p53and Bcl-X_L_, has been studied by Bharatham *et al.* [93], finding that the residues Glu96, Phe97, Arg100, Phe105, Leu108, Val126, Leu130, Ala142 of Bcl-X_L_ play important roles in the interaction with p53 TAD, in which the three conserved hydrophobic residues (Phe19, Trp23 and Leu26) were found to be anchored into three hydrophobic pockets of Bcl-X_L_.

It has been observed that the copper-containing protein azurin (AZ), with electron transfer activity in Pseudomonas aeruginosa, binds p53 protein via either the TAD or the DBD and enhances the structural stability of p53 to activate its tumor suppressor function [31–33]. To explore the molecular interaction mechanism of the p53-AZ complex, Taranta *et al.* [94] modeled the complex structure of TAD peptide of p53 (residues 17–56) and the bacterial protein AZ, performing MD simulations followed by binding free energy estimation, and validating the predicted complex structure by mutagenesis studies. The short-range hydrophobic interactions and a lot of hydrogen bonds were proposed to be the main driving force for the stabilization of the complex.

These results shed further light on different features of recognitions between p53 TAD and protein partners and maybe helpful for rational design and development of new potent and selective inhibitors which can restore the function of p53.

#### 2.2.2. Protein-Protein Interactions Located in DBD and OD of p53

The three-dimensional structures of the entire DNA-binding domain [15] and oligomerization domain [16] have been determined (Figure 1B,C). Several studies found that the metal cation located in the DBD is important for aggregation and sequence-specific DNA binding [95,96]. DBD is often unstable and contains most of the inactivating single missense mutations occurring in human tumors [12].

The protein Azurin (AZ) has been demonstrated to enhance p53 stability via binding to not only TAD but also the p53 DBD. Computational methods including molecular docking, MD simulations and free binding energy estimation by MM-PBSA, were carried out to understand the interaction between AZ and p53 DBD [97]. Recent research by Santini *et al.* [98] focused on the interaction between p28 (the peptide fragment of Azurin, residues 50 to 77) and the p53 DBD peptide using computational docking coupled with MD simulations and binding free energy estimations. The p28 fragment should retain the cellular penetration ability and anti-proliferative activity of the whole protein. Both the folded and the unfolded structures of the p28 peptide have been found to form a stable complex with the p53 DBD by favorable low binding free energy, high shape complementarity, predominant polar interfaces and hydrogen bond interactions.

Tetramerization of the p53 protein is essential for its function, such as for DNA binding, protein-protein interactions, post-translational modifications, and p53 degradation [99]. The small oligomerization domain (OD) comprising one β-strand, one tight turn and one α-helix (Figure 1C), is responsible for the formation of the stable native tetramer, which is generally formed by a dimer (consisting of an anti-parallel β-sheet and an anti-parallel helix-helix interface) of dimers [16,100,101].

Dimerization of the p53 OD and unfolding of the tetramerization domain have been studied by running MD simulations [102,103], revealing that formation of the folding nucleus precedes and appears to be necessary for the formation of hydrogen bonds in the β-sheet as well as other inter-monomer contacts. The tetramerization is stabilized by hydrophobic interactions while disruption of the native tetrameric hydrophobic core results in the beginning of unfolding.

Moreover, single mutation R337H can destabilize the tetramer and inactivate its function [104,105]. A strong salt bridge formed by Arg337 and Asp352 from another monomer at four equivalent sites, is very important to the overall tetramer stability [100]. Interestingly, a fluid salt-bridging cluster of Arg337, Arg333, Glu349 and Asp352 was observed during the MD simulations of wild type p53 and the R337H mutant at several different pH and salt conditions [106], suggesting that these four residues contribute significantly to the tetramer stability. The mutation R337H still interacts with Asp352, but loses much of its interaction with Glu349. When His becomes deprotonated at high pH, the interaction between this deprotonated residue and Asp352 is also lost, which was considered the reason for strong destabilization of mutation R337H observed at high pH.

Additionally, published observations [107–112] suggest that S100B can binds to the tetramerization domain of p53 when it is exposed in lower oligomerization states, and S100B not only inhibits p53 tetramer formation but also promotes disassembly of the p53 oligomers. These findings imply that reduced the levels of S100B in tumors could restore p53 functions and contribute to cancer prevention. However, up till now its molecule mechanism of the interactions between S100B and the p53 OD has not been clearly understood and will require further research.

#### 2.2.3. Protein-Protein Interactions Located in CTD of p53

The C-terminal region is subject to extensive post-translational modifications including acetylation, methylation, phosphorylation and ubiquitination that regulate function and cellular protein levels.

Unlike other proteins that bind to or modify the CTD of p53, proteins that activate the tumor suppressor, inactivation was observed for S100B, a member of the best characterized proteins of the S100 protein family involved in cell cycle progression and cell differentiation [62,63,107–112]. Researchers have reported that the tumor suppressor protein p53 interacts in a calcium-dependent manner with dimeric S100B [107], and consequently the PKC-dependent phosphorylation of p53 in its CTD is suppressed by protein-protein interactions [62,63,111,112]. Interestingly, S100B not only binds to the p53 tetramerization domain and controls its oligomerization state, but also binds to the p53 CTD, which is unstructured in its native form, as revealed by nuclear magnetic resonance (NMR) spectroscopy. It forms an α-helix secondary structure that spans residues 377–387 when binding to the Ca^2+^-bound S100B(betabeta) partner (PDB ID: 1DT7) (Figure 1D) [19]. Two Ca^2+^ at the loop regions (residues 20–25, and 61–66) are bound to each monomer of the homo-dimeric S100B in solution and trigger the binding of S100B with p53. It is believed that the p53 CTD recognizes protein S100B through a “fly-casting”-like process [113]. The S100B sterically hinders sites of phosphorylation (at positions Ser376 and Thr377) and acetylation (at position Lys382) on p53 which are crucial for the activation of transcription [19,114].

The hydrophobic surface of S100B is mainly formed by the helix 2 (residues 29–39), the hinge region (residues 40–49), the helix 3 (residues 50–60), and the helix 4 (residues 70–88) (Figure 1D). The comparative structural studies reveal that S100B undergoes a conformational change upon binding Ca^2+^ and generates the hydrophobic surface through changing the orientation of helix 3 and helix 4 and placing helix 3 nearly perpendicular to helix 4 and elongating the helix 4 by one turn. This conformational change results in the formation of several favorable hydrophobic and electrostatic interactions and promotes its interaction with the target p53 protein as well as disassembly of the p53 oligomerization [19,62,107,111,115]. Since p53 protein functions as a tumor suppressor only in its tetrameric form, the binding of S100B disrupts the tetramerization equilibrium of p53 and thus closely relates to the genesis and progression of malignant tumors [116–118]. Therefore, it is necessary to develop high-efficient and low-toxic ligands to block the p53-S100B interaction in order to protect p53 from S100B-dependent down-regulation and restore the tumor suppressor function. Several theoretical studies were performed to understand the p53-S100B interaction [119–121]. In this section, latest molecular dynamic simulation studies are discussed.

Recently, the binding process between the p53 CTD and five partners including the S100B was investigated using MD simulation by Allen *et al.* [120]. Each complex in addition to the p53 CTD fragment alone and the S100B alone were simulated in triplicate for 100 ns with GROMACS. When bound to the S100B, the α-helix structure of the p53 CTD comprising residues 380–387 underwent little change from the starting structure in the first and third replicates during each 100 ns simulation while the α helix was found to form a turn in the second replicate. When unbound to the S100B, the secondary structure of p53 CTD changes from α-helix to other forms, most notably β-sheets and turns. The RMSD of the S100B and p53 CTD fragment fluctuates less in bound state, especial for the p53 CTD compared with its free state in solution. The hinge region (residues 40–49), helix 3 and helix 4, shaping the p53 CTD binding site, all fluctuate less but the hinge region located in calcium binding site (residues 20–25) fluctuated more for the p53 CTD fragment-bound state. The observation was in agreement with the structural research [19] that S100B underwent a large conformational change in helix 3 upon binding Ca^2+^ and this change was advantageous to the opening of the large hydrophobic core, including S100B residues Met79, Val80, Leu44, and Val56, which were necessary for the binding of p53 CTD. The configurational principal components analysis (PCA) which describes large collective motions, also confirmed a stabilization effect of S100B on the p53 CTD fragment upon binding to the S100B. The electropositive p53 CTD residue Arg379 forms stable hydrogen bonds with His42 and Glu45, so does Lys382 with Glu86 and Phe88. Other residues His368, Ser378, and Lys381 also form many favorable electrostatic contacts with S100B residues Glu45, Glu46, Lys48, Glu49, Glu51, Lys55, and Glu89. In addition, the van der Waals contacts formed by Arg379, Lys382, Leu383, Met384 and Phe385 of the p53 CTD fragment and S100B hydrophobic core defined by residues Leu44, Val52, Val56, Met79, Val80, and Phe87 stabilize the protein-protein complex. The author Allen [120] offered a hypothesis about the specific α-helix conformation adopted by the p53 CTD peptide when bound to S100B, which enables alignment of the hydrophobic residues (Leu383, Met384, and Phe385) toward the hydrophobic binding site. The Ser376 and Thr377 of the p53 CTD were believed to be buried against the surface of S100B, and the MD simulations confirmed that Thr377 tends to stay buried and forms hydrogen bonds with residues Glu45, Glu46, and Glu49, but Ser376 becomes solvent exposed.

To investigate the structural effects of a novel single point mutation (G389E), MD simulations of the free wild type (WT) and mutant (G389E) peptides corresponding to the extreme CTD, in solution and in complex with S100B were carried out in 2010 by Pirolli and co-workers [121]. In these studies, the helical stability of the CTD fragment of p53 was analyzed. The p53 α-helix G389E mutant was found to be unfolded rapidly in the absence of S100B in solution and WT peptide gradually loses its helical conformation which confirmed that the free p53 CTD exists in a disordered structure. The G389E mutation has negligible effect on the phosphorylation (residues Ser376, Thr377) and acetylation (residue Lys382) sites when it is free in the solution. The p53 CTD peptide is very flexible when bound to S100B, especially in the G389E complex. The mutant residue increases its α-helix content, while S100B is stable in both simulations. The α-helix of WT p53 was found to be gradually lost and undergo a large conformational change and form a closed peptide at the end of the simulation. Snapshots from simulations showed that the negatively charged residue Asp391 and Asp393 alternatively interact with positively charged residues Lys370, Lys372 and Lys373. The persistent direct salt bridge between Asp391 and Lys373 and one hydrogen bond between the Glu388 and Thr377 contributed to this conformational change. In the presence of this novel G389E mutation, the binding interface is significantly altered, and Lys382 and Lys370 form a direct salt bridge with Thr82 and Glu89 located in helix 4 of S100B, respectively, thus the normal function of p53 is prevented.

It has been known the acetylation of Lysine of p53 is necessary to destabilize the p53-MDM2 interaction and facilitate p53-mediated stress response [122]. The activity and stability of p53 is also regulated by the lysine acetyltransferase and transcriptional coactivator cAMP response element-binding (CREB)-binding protein (CBP)/p300 [123–125]. Modulation of acetylation-mediated interactions by small inhibitors may be a new approach for anti-cancer drug design and research [125–128]. NMR structural analysis reveals that bromo-domain of CBP (residues 1081–1196) binds specifically to the acetylated lysine 382 in the CTD (residues 367–386) [39]. The p53-CBP interaction was investigated by MD simulations and the energetics of the binding complex was also estimated by Free Energy Perturbation (FEP) [120,129].

Allen *et al.* [120] observed that a moderate change of bend conformation occurred along the middle of the chain of p53 CTD fragment in the complex during MD simulation. Drastic changes in the N- and C-terminal ends cause them to come together as β-strands to form a stable and persisted anti-parallel β-sheet. These occurred after 20 ns simulation for the free CTD fragment in solution. On the other side, the opening and closing of the hydrophobic binding site between the ZA loop (residues 1115–1138) and the BC loop (residues 1170–1190) in the bromo-domain represented 23.8% when bound to p53 CTD and up to 58.4% of the total motion for CBP protein in the absence of the p53 CTD according to the PCs analysis. In the initial structure, only six residues (381–386) located in the C-terminal of p53 CTD are in close contact with CBP, while the rest of the fragment quickly folds over and the polar residues His368, Ser371, Lys373, and Gln375 of p53 CTD interact with Asp1124 and Asp1127 of the CBP during the MD simulation. The residue AcLys382 consistently remains to be inserted into the hydrophobic cavity which is formed by Val1115, Ile1122, Tyr1125, Tyr1167, Val1174, and Phe1177 of CBP. The FEP simulations of the acetylated and non-acetylated peptides in complex with CBP bromo-domain showed that the relative contribution of the acetyl group to p53 binding is 4.8 ± 0.5 kcal/mol [129], which is a major portion of the binding free energy and in excellent agreement with experimental results spanning a range of 4.3–4.9 kcal/mol [130]. Phe385 and Lys386 were found to form contacts with Arg1112. Thus hydrophobic contacts are the dominant factor in the binding of CBP bromo-domain with p53 CTD. In addition, the salt bridges between Arg379 and Asp1124, Lys386 and Glu1105, and the H-bonding between AcLys382 and Tyr1167, AcLys382 and Asn1168, Leu383 and Ser1172 also contribute to the interaction [129]. All in all, it is clear that the AcLys382 plays an important role in the interaction between p53 and CBP.

Sirtuin-2 (SIRT2), a NAD^+^-dependent histone deacetylases, is crucial in cell cycle regulation [131–134], specifically binds and deacetylates p53 protein and functions in transcriptional silencing processes [135,136]. The X-ray co-crystal structure shows that the Lys382 acetylated p53 CTD peptide forms a β-strand from residues 379 to 387 (PDB ID: 1MA3), where the acetyl-lysine side chain inserts into a conserved, largely hydrophobic tunnel when it binds to a Sirtuin protein (Sir2) [38]. This β-strand of the acetylated peptide binds in the cleft and forms a stable β-sheet along with two other β-strands of Sir2. The binding process between the p53 CTD and Sir2 was investigated by MD simulation [120]. The results show that acetylated p53 CTD fragment exhibits very little secondary structure, with only a turn formed around residue Lys 381 in two of the three replicate simulations when bound to Sir2. However, the unbound acetylated p53 CTD fragment adopts many different ordered conformations, including a discontinuous turn and most notably a very short sheet-turn-sheet structural motif. The RMSD analysis reveals that the acetylated p53 CTD fragment becomes stable when bound to the Sir2. In the absence of p53 CTD, the p53 CTD-Sir2 interface (residues 45–60 and residues 150–175 in the Sir2) and most notably hydrophilic and acidic residues near the hydrophobic binding site of Sir2 fluctuate more. The backbone atoms of His380, AcLys382, Leu383 and Phe385 of the p53 CTD form persistent contacts with Gly166, Glu167, Leu169, Val195 and Tyr197 of Sir2 in all three replicate simulations. Van der Waals interactions between the AcLys382 of p53 CTD fragment and His118, Val63, Phe165, Leu169, and Val196 of Sir2 were found to be the major contributors to the interaction energy. Among all residues, the AcLys382 retains its position and plays the largest role in the acetylated p53 CTD-Sir2 interaction. Lysine acetylation of p53 is also required for its activation as a transcription factor.

Several studies have established that lysine methylation, like protein histone lysine methyltransferases KMT5 (SET9) methylates p53 at specific C-terminal lysines, and is a novel mechanism for modulating of p53 function [23,40,137,138]. In order to understand the underlying mechanisms, MD simulations were preformed to investigate the details of this interaction [120,139].

The crystal structure of the SET9 in complex with a mono-methylated (MeLys372) p53 CTD fragment (residues 369–374) was solved at 1.75 Å and used as the initial structure for MD simulations. It is different from the clear secondary structure when bound to S100B [19] or Sir2 [38], the C-terminal residues of p53 CTD were not solved and only 6-mer fragment left, it lacks any ordered secondary structure. According to Allen’s study [120], only a bend centered at Ser 371 was detected when the p53 CTD in complex with SET9, whereas a sporadic turn and bend (residues 370–374) in the absence of SET9. The increased fluctuation was observed in the absence of the p53 CTD for the two important hydrophilic and acidic residues Asp256 and Thr266 in p53 CTD binding. PCA suggested a single vector corresponding to the opening and closing of the CTD binding site, described 72.0% of the overall motion in Set9 when it is free, but dropped to be only 38.3% when bound to p53 CTD. The MeLys372 inserts into a hydrophobic channel partly formed by the residues Val255 and Leu267. The residues Asp256, Thr266, and Ser268 of Set9 form stable hydrogen bonds with the p53 CTD fragment.

It is generally accepted that water plays an important role in the methylation process. Another study [139] was reported recently, finding the channel that surrounds p53-MeLys372 was not suitable for water movement, but a new channel composed of Gly292, Ala295, Tyr305 and Tyr335 was large enough for water moving in and out of the active site. The results indicated that SET9 is able to dimethylate p53-MeLys372.

Phosphorylation also modulates p53 function. The crystal structure of a complex containing a 9-mer p53 CTD fragment (residues 378–386) and cyclin A was solved at 2.24 Å [41]. To investigate the interactions between the two proteins, MD simulations were preformed [120]. The results pointed that the structure of the p53 CTD fragment became stabile when bound to cyclin A. The cyclin A forms a small hydrophobic binding pocket, in which Leu253 interacts with the hydrophobic residues of the p53 CTD. The residues Glu220, Glu224 and Asp283 of cyclin A contact with Ser378, Arg379, His380 and Lys381 of the p53 CTD comprising an important electrostatic contribution, Ile213, Leu214, and Leu253 of cyclin A interact with Leu383 and Phe385 of the p53 CTD creating the van der Waals contribution.

In summary, site specific post-translational modifications of acetylation, methylation and phosphorylation in CTD play an important role in the function of regulation of p53.

### 2.3. Small Molecule and Peptide Inhibitors Computational Modeling and Design

Recently, inhibition of the interaction between MDM2/X and p53 using non-peptide small-molecule or peptide inhibitor has received increasing attention and has been widely studied experimentally and computationally. For the well-defined structure of MDM2/X, inhibitors prefer to mimic p53 rather than MDM2/X, mainly mimic the side chain of three hydrophobic residues Phe19, Trp23 and Leu26, and the Leu22 which is also subsequently found to play a vital role in the p53-MDM2/X interaction, in the hope of gaining higher binding affinities than p53 TAD peptide [140]. The structures of p53-MDM2 and p53-MDMX (Figure 3) showed that the interactions of Phe19 and Trp23 of p53 are highly similar, but Leu26 is different. Besides, the three residues Leu54, His96 and Ile99 in MDM2 are different from the equivalent residues Met53, Pro95 and Leu98 in MDMX. These differences are possibly attributed to the variant binding of p53 peptide to MDM2 and MDMX.

Hu *et al.* [141] carried out molecular docking and MD simulations to explore the dynamic behavior of MDM2 with a set of six non-peptide small-molecule inhibitors, extracted from the compounds designed by Ding *et al.* [142], and estimated the relative binding affinities by MM-PBSA. These inhibitors have a similar structure including 4 groups. For the inhibitor 8, G1 represents for 6-chloro-2-oxo-indole group, G2 for 3-chloro-2-fluoro-phenyl group, G3 for 2,2-dimethylbutane group and G4 for 2-morpholin-4yl-ethylamine group. The study showed that the van der Waals energies (−30 to −44 kcal/mol) are the dominant driving force for each complex, which indicated that the shape complementarity is the major contribution to the affinities. The MD simulations also showed that G1, G2 and G3 group in averaged structure mimic the Phe19, Trp23 and Leu26 residues in p53, respectively, with the hydrogen bond well kept. However, the G4 group does not act as the residue Leu22 of p53, interacting with the residues Phe55, Gln59 and Met62 of MDM2 by C–H…π and C–H…O interactions.

The inhibitory mechanisms of four small molecules on the MDM2-p53 interaction were investigated in our lab by 10 ns MD simulations and MM-GBSA analysis [143]. The binding free energies were −15.59, −15.63, −14.21 and −12.49 kcal/mol for IMZ, DIZ, YIN and K23 to MDM2, respectively. It can be seen that the estimated rank was in excellent agreement with the experimental rank. The van der Waals energy (spanning −37 to −45 kcal/mol) was the dominant factor for the binding of the four inhibitors among the binding free energy components. The R1, R2 and R3 (R4) groups of these inhibitors insert into three hydrophobic sub-pockets of MDM2, producing CH-π and π-π interactions, which are favorable for the stable binding. In this study, the six residues Leu54, Gly58, Ile61, Met62, Val93 and His96 in MDM2 were found to play important roles in hydrophobic interactions with the four inhibitors.

The inhibitory activity of 1,4-benzodiazepine-2,5-diones (BDPs) and *N*-Acylpolyamine (NAPA) derivatives to HDM2 was investigated by Wang *et al.* [144] through 3D-QSAR and MD-based docking. For BDPs, the key residues are Gln24, Lys51, Gly16, Ser17, Leu54, Gln72, Gly58, Leu57, Val93, Phe55 and His73. The small electronegative groups at R1 and R3 regions mainly involved H-bonds formation, while large groups at R2 and R4 regions are favorable for BDP’s inhibitory activity. On the other hand, for the NAPA-protein complex, the key residues are Glu25, Thr26, Tyr100, Tyr104, His96, Leu54, Met50 and Phe55. Both huge and electropositive groups in B ring and A ring, and small groups at region P are to the benefit of NAPA derivatives inhibitory activity.

The p53-mimic peptide PMI (sequence TSFAEYWNLLSP, Figure 2D) has higher affinity than the p53 (the corresponding sequence ETFSDLWKLLPE) to the binding of MDM2. In order to explore the role of each residue in this peptide, Liu *et al.* [145] preformed multiple short simulations in explicit water environment of each alanine mutation. The binding free energies from the short MD simulations were in good agreement with that from 50 ns MD simulations. The F3A, Y6A, W7A, L10A mutants of PMI incur large loss of both van der Waals (8, 9, 12 and 6 kT for F3A, Y6A, W7A and L10A, respectively) and electrostatic interaction energies (12, 11 and 5 kT for F3A, Y6A and W7A, respectively) than other mutants.

The MD simulations followed by MM-GBSA method were performed to explore the binding mechanisms of peptide and small molecule inhibitors to MDMX (PMI-MDMX and WW8-MDMX complexes) by Cheng *et al.* [146] (Figure 2B,D). The non-peptide WW8 induces high flexibility, in particular, the α-helix in the C-terminal of MDMX is more obvious, and a stronger anti-correlate motion than peptide PMI for MDMX in most regions except the loop L2. Compared to the initial structure, the side chain conformation of residue Tyr99 was changed to generate a steric clash in both simulations. The binding free energies estimated by MM-GBSA are −15.49 and −9.26 kcal/mol, and van der Waals energies are −62.71 and −45.90 kcal/mol for PMI-MDMX and WW8-MDMX, respectively. The van der Waals energies are the major favorable contributors to the inhibitor-MDMX bindings. For its extra interactions with Lys50, Tyr66, Gln71 and His72, the peptide inhibitor PMI generate much stronger electrostatic and van der Waals interactions than non-peptide inhibitor WW8 with MDMX. Six common residues Met53, Ile60, Met61, Tyr66, Val92 and Leu98 are located in the hot spots of the interface between the inhibitors and MDMX determined by computational alanine scanning. These results lead to a conclusion that the hydrophobic CH-CH, CH-π and π-π interaction play important roles in the inhibitor-MDMX binding, and the peptide inhibitors stabilize the inhibitor-MDMX complex.

Though the two proteins MDM2 and MDMX share structural homology, their p53 binding sites are not exact same, and some inhibitors (like nutlin) show different affinity to MDM2 and MDMX. Consequently, to identify MDM2/MDMX dual-inhibitors opens a new era of anti-cancer drug development. Barakat *et al.* [147] conducted such a study by ensemble-based virtual screening to identify effective dual-inhibitors which could simultaneously disrupt the p53-MDM2/X interactions. Their results confirmed the three MDM2-inhibitors, Nutlin3, MI-219 and TDP, have low affinities to MDMX.

The first non-peptide small-molecule used to block p53-MDM2/X interactions was nutlin (Figure 2A). The different binding modes of p53 peptide and nutlin to MDM2 and MDMX were investigated by Joseph *et al.* [148] using MD simulations. Residues Glu69, Lys70 and Val93 in MDM2 and Gln69, Glu70, Asp79 and Asp94 in MDMX displayed the highest fluctuations. The p53 peptide appears to stable the two proteins so that the p53-bound states display less flexibility than their apo and nutlin-bound states. A longer helix was formed for p53 peptide when bound to MDM2 than to MDMX, for a salt bridge between Glu17 of p53 with Arg65 in MDM2 was formed. The hydrogen bond between Trp23 (p53) and Leu54 (MDM2) and the intra-molecular hydrogen bond between Glu17 (MDM2) and Ser20 (MDM2), in particular, the salt bridge between Glu17 and Arg65 plays an important role in maintaining the stability of complex. The hydrogen bond between Trp23 (p53) and Leu54 (MDMX) and the intra-molecular hydrogen bond between Phe19 (MDMX) and Gln17 (MDMX), are retained with the occupancy of 48% and 66% respectively during the simulations. However, the corresponding salt bridge Glu17-Arg65 in MDM2 could not form in MDMX for this Arg65 was replaced by Gln64. The binding free energies were estimated to be −6.4 and −3.7 kcal/mol for p53-MDM2 and p53-MDMX, respectively. Since the van der Waals interactions were similar, the driving force for the binding of p53-MDM2 was easily identified due to stronger electrostatic interactions than p53-MDMX. When bound to nutlin, Leu54 in MDM2 is replaced by the larger Met53 in MDMX, the binding pocket of MDMX is changed to be smaller than that in MDM2, resulting in reduced electrostatic and van der Waals interactions for MDMX-nutlin. The larger entropic penalty for p53 upon the binding leads to a lower affinity of p53 than nutlin for MDM2. The binding free energies were estimated to be −17.9 and −0.3 kcal/mol for MDM2 and MDMX in the nutlin-bound state, respectively. MDM2 binds both p53 and nutlin while MDMX interacts with nutlin with extremely low affinity.

Lu *et al.* [149] studied the pyrrolopyrimidine-based α-helix mimetic dual inhibitors of MDM2 and MDMX, including an active compound 3a and one inactive compound NC-1, using molecular docking and MD simulations followed by MM-PB/GBSA calculations. The R1, R2, and R3 groups of the pyrrolopyrimidine scaffold compound 3a could mimic the spatial orientation of the side chains of Phe19, Trp23, and Leu26 in p53 and their interactions with MDM2/X, respectively. However, the R1 group of NC-1 is a methyl sulfonyl moiety that could not mimic the side chain of Phe19 of p53. The compound 3a has similar binding affinity for both protein MDM2 and MDMX than that of NC-1, which departs from the active site of MDMX during MD simulations, with the binding free energies of −15.16(PB)/−14.74(GB) −3.01(PB)/−2.23(GB) and −13.97(PB)/−14.24(GB) kcal/mol for MDM2-3a, MDM2-NC-1 and MDMX-3a respectively. The residues Leu54/Met53, Ile61/Ile60, Gln72/Gln71, His73/His72, and Val93/Val92 for MDM2 and MDMX, which locate in the Phe19 sub-pocket of p53 have made outstanding contributions to the binding in the hydrophobic interface cleft. This result also proved that the critical residue Phe19 is indispensable for the interaction between p53 and MDM2/X. The interactions between those two inhibitors and MDM2/X are dominated by the large van der Waals energy (−47.69, −34.35 and −48.87 kcal/mol), which indicates that the shape complementarity is important for designing high affinity inhibitors.

Another study to probe the difference in binding mechanisms of peptide (pDI6W, sequence LTFEHWWAQLTS) and non-peptide inhibitor (K23, with four aromatic groups) to MDM2/X was also investigated by our lab [150] (Figure 2C,E). The dynamic behavior analysis suggested that the inhibitor-MDM2 is much more stable than inhibitor-MDMX complex. Tyr99 in MDMX undergoes a larger conformational change for K23-bound than pDI6W-bound. The predicted binding free energies are −21.93, −19.74, −16.81 and −14.89 kcal/mol for pDI6W-MDM2, pDI6W-MDMX, K23-MDM2 and K23-MDMX complex, respectively, which agree well with the experimental rank. The van der Waals energies were found to dominate the bindings of the inhibitors in the hydrophobic cleft of MDM2/X. The peptide inhibitor pDI6W generates much stronger electrostatic and van der Waals interactions with the two proteins than the non-peptide inhibitor K23. The binding mode analysis for inhibitor-MDM2 complex revealed that four residues Tyr67, Met62, Ile61 and Gln72 form the first hydrophobic sub-pocket (Phe19 sub-pocket); Leu54, Gly58 and Val93 form the second hydrophobic sub-pocket (Trp23 sub-pocket); Leu54, Val93, His96 and Ile99 form the third hydrophobic sub-pocket (Leu26 sub-pocket). For the inhibitor-MDMX complex, residues Ile60, Met53, Tyr66 and Gln71 enclose the first hydrophobic sub-pocket; Met53, His54, Gly57 and Val92 shape the second hydrophobic sub-pocket; Met53, Val92, Pro95 and Leu98 shape the third hydrophobic sub-pocket. According to the computational alanine scanning analysis, it is clear that the π-π, CH-π and CH-CH interactions produced by eight residues (Lys51, Leu54, Ile61, Met62, Tyr67, Val93, His96 and Ile99) in the pDI6W-MDM2 complex, four residues (Leu54, Ile61, Val93 and Ile99) in the K23-MDM2 complex, six residues (Lys50, His54, Ile60, Met61, Tyr66 and Val92) in the pDI6W-MDMX complex and four residues (Met53, Ile60, Val92 and Leu98) in the K23-MDMX complex, play a key roles in the binding of the inhibitors in the hydrophobic cleft of MDM2/X.

In some sense peptide inhibitors have an advantage over non-peptide small molecules in targeting protein-protein interaction interfaces since they can be finely tuned to be much more specific. However, peptide inhibitors are usually unstable and subject to proteolytic degradation. In order to address this problem, the “peptide stapling” strategy using an all-hydrocarbon cross-link (staple) was applied to generate a series of stabilized alpha-helix of p53 (SAH-p53) cyclic peptides [151], in which the key interacting residues were kept and the amphiphilic α-helical structure was reinforced. Such SAH-p53 peptides exhibit significantly high affinity for MDM2/X in contrast to the corresponding native p53 peptide, and improve the cell permeability and increased helical stability compared to the peptidomimetics, therefore, they are becoming a new trend of anti-cancer drug development. The structure of the MDM2 bound to a stapled p53 peptide was determined recently [61] (Figure 2E). There have been some stapled peptide inhibitors, with increased helicity and cellular uptake without apparent cytotoxicity, are also reported [152–154].

To explore the structural and dynamic stability of the hydrocarbon cross-links stapled peptides, Guo *et al.* [155] studied 10 stapled α-helical peptides of the 16-residue of the p53 TAD fragment over a range of temperatures in solution by all-atom MD simulations. The overall propensity to form a-helical structure of stapled peptides was found to exhibit substantial variations, depending both on the relative position of the stapling and the specific residue substitutions. In order to understand their effects on the inhibition of MDM2/X, Joseph *et al.* [156] reported novel interactions of the staples with the target proteins by performing on all-atom MD simulations on the complexes of 9 stapled peptides to MDM2 and 2 stapled peptides to MDMX. The study confirmed that good binders extend the helicity in solution and increase the affinity when bound to MDM2 for the extensive interaction between hydrocarbon staples and the MDM2 surface. In addition, the contributions of stapled peptides are equivalent to Phe19 and Trp23 of p53 peptide. The stapling site, charge quantity and distribution, and overall hydrophobicity are important for the inhibitory activity. These studies provide new insights into the anti-cancer drug design of stapled peptides and the development of potent inhibitors preventing p53 from interacting with target proteins.

Like MDM2, S100B is activated at the transcriptional level by active p53 and then subsequently negatively regulates p53 function as a tumor suppressor via feedback loop control [112,157,158]. As one of the most effective ideas to develop anti-cancer drugs, there has been some researches focusing on the identification, characterization, design and *in vitro* screening of small molecule inhibitors of the interaction between calcium-dependent S100B and p53 tumor suppressor [64,159–161]. In addition, nuclear magnetic resonance and X-ray crystallography studies have been reported about the structures of three small molecules (SBi132, SBi1279, and SBi523) and these three molecules were found to bind in distinct locations and orientations within the hydrophobic binding cleft of calcium-bound S100B [162]. The strategy of combining computer aided drug design method with NMR spectroscopy and X-ray crystallography is being used to identify inhibitors of the p53-S100B interaction. Several computational studies about the ligands with the potential to block the binding of p53-S100B have been published [163,164].

Whitlow JL and co-workers [163] studied the interaction between p53 and S100B (PDB ID: 1DT7) using MD simulations followed by the MM-PBSA method. Initially, organic molecules were identified as potential inhibitors to block the p53-S100B interaction using the LUDI program implemented in Cerius2 package and ZINC database, and then 14 ZINC compounds were selected for the S100B-ligand binding affinity calculation by MM-PBSA. The RMSD of the p53-S100B complex from MD demonstrated that the C-terminal peptide of p53 is very flexible whereas the S100B structure stays very stable. The same results also appeared in the subsequent MD studies [120,121], indicating that the α-helical structure of C-terminal peptide of p53 gradually loses its secondary structure during simulation. The analysis of the interaction between S100B and p53 showed that the van der Waals interaction is very weak and the electrostatic interaction dominates the binding of S100B with p53 CTD. This indicates that the residues Glu45 and Glu46 of S100B have the strongest interaction with p53. For the S100B-ligand systems, the 14 ZINC compounds were found to interact with S100B at residues Glu45, Glu46, Leu44, Val52, Lys55, Val56, Thr59, Phe76, Met79, Val80, Ala83 and Phe87, displaying the similar binding modes and interactions as p53 with S100B, and therefore it was expected that they have the potential to prevent S100B from binding to p53 [163].

Another paper exploring ligand blockage of the interaction with p53 [164] was published in 2009. Three compounds with the highest binding affinity to the S100B and the highest potential to block the protein interaction of S100B and p53 were selected from the NCI small molecular library using the Glide docking program. Based on the docked structures, it could be seen that the hydrophobic aromatic rings of the three selected compounds directly contact with S100B in the hydrophobic binding site mainly formed by the helix 2, 3, and 4. Besides, the hydrogen bonds between compounds and residues Glu45, Thr59, Gln71, and Lys111 of S100B were formed. MD simulations showed that the ligand induces the separation of p53 from S100B by about 2Å and the decreases of the binding affinity of S100B and p53 by ~8.5–14.6 kcal/mol compared to the original p53-S100B complex. Based on the binding free energy calculation done by MM-PBSA, the van der Waals energy could be the major driving force for the binding of the complexes of p53-S100B with or without the three ligands and the complexes of S100B-ligand. It could be perceived that the three compounds with hydrophobic aromatic rings propelled p53 away from S100B, and they are able to bind to free S100B and form stable S100B-ligand complexes with the binding affinities no weaker than p53. Therefore the availability of p53 is able to restore its biological function as a tumor suppressor. Design of compounds by effectively blocking p53-S100B interactions has been regarded as a new promising approach to the development of anti-cancer drugs.

## 3. Outlook

It is well known that p53 exerts its tumor suppressor function through a series of interactions of independently folded and intrinsically disordered functional domains. The full-length three-dimensional structure of this protein has not been determined to date. The unstructured TAD and CTD are especially regarded as the important modulatory domains for core interactions with specific sites for DNA binding.

Mutation, post-translational modifications and physiological interactions of vital residues in p53 with numerous negative regulator protein partners have a certain influence on the biological function of p53. At the same time, these negative regulator protein partners also interact with each other, such as MDM2-MDMX interaction [165,166] and MDM2-S100B interaction [167]. In addition, several proteins can bind p53 with more than one domain. All of these factors combine to create a complex regulation mechanism and network for p53 function.

Inhibitor design primarily mimics key interactions between p53 and protein partners, in order to achieve the purpose of releasing p53. Restoring endogenous p53 functions by the disruption of p53-protein interactions using peptide or non-peptide small molecule inhibitors holds a lot of promising strategies for anti-cancer drug design and development. Generally speaking, peptide inhibitors have an advantage over non-peptide small molecule inhibitors in targeting protein-protein interaction interfaces. Nevertheless, peptide inhibitors are unstructured in solution and highly susceptible to proteolytic degradation *in vivo* and therefore unsuitable for potential drugs. The “peptide stapling” strategy, using an all-hydrocarbon staple to enhance the stability and improve the cell permeability, may open a new era in the design and development of anti-cancer drug. Furthermore, traditional Chinese medicine and natural products attract a lot of attention [168–173] for their inhibitory activity of tumor growth and ability to induce apoptosis through restoring the p53 function to some extent. This research provides some trains of thought on the discovery and development of novel anti-cancer drugs.

With a better understanding of the interaction mechanism between p53 and its protein partner, it is possible to achieve efficient drug design. Today’s MD simulations mainly adopt the classical molecular mechanics force fields, which rely on the representation of atomic electrostatic properties using fixed-point charge models. However, these crude models neither fully describe the real molecular electrostatic distribution, nor consider the influence of electrostatic polarization. The estimated binding free energies from MD simulations are often artificially high compared to measured values. Previous MD simulation studies suggested that electrostatic interactions and electrostatic component of solvation free energy provide a dominant role in p53-protein interactions. From the molecular simulation point of view, the polarizable force field is one of the hot topics considering the accurate electrostatic and polarization effect, which may be more suitable for this interaction study, thereby contributing to the rational development and design of new anti-cancer drugs.
